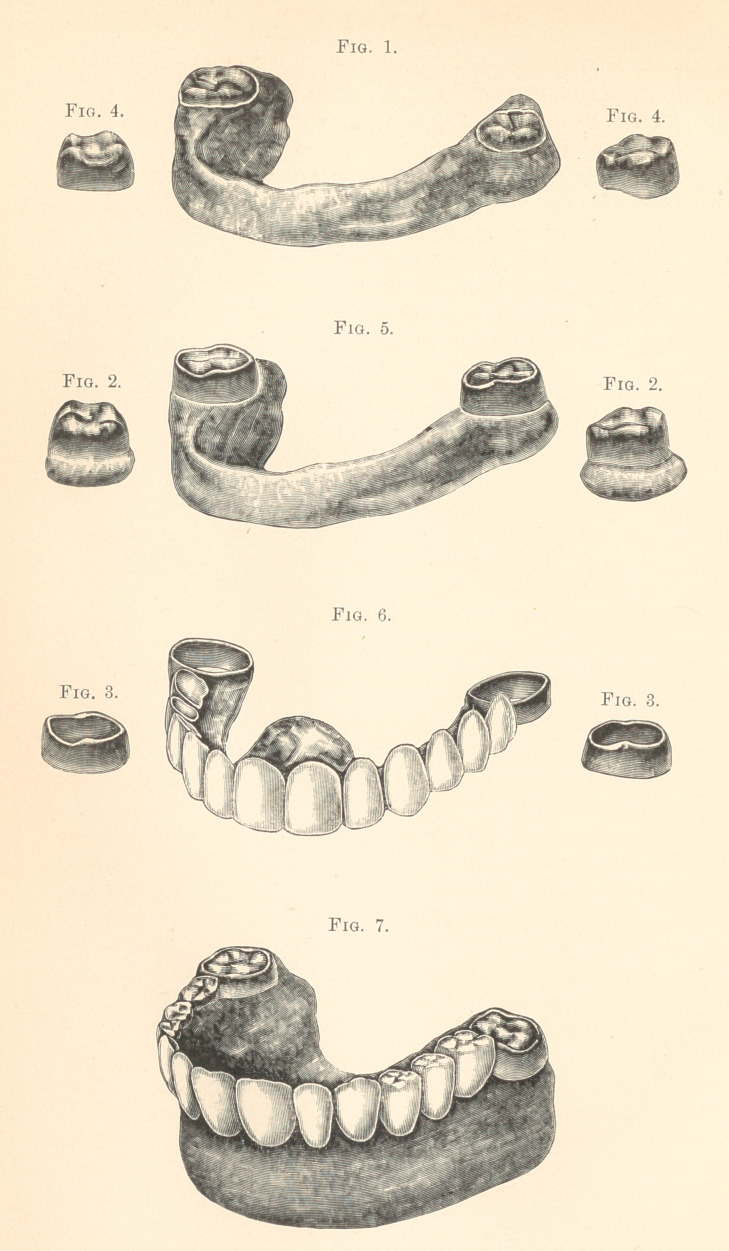# Crown- and Bridge-Work

**Published:** 1892-07

**Authors:** C. M. Richmond

**Affiliations:** New York


### International Dental Journal.

Vol. XIII. July, 1892. No. 7.

### Original Communications.[1]

### CROWN- AND BRIDGE-WORK.[2]

BY DR. C. M. RICHMOND, NEW YORK.

[The present article is preliminary to a series of papers in which it is proposed to explain the various processes adopted to facilitate the preparation of this form of denture. The text will be fully illustrated to enable the reader, by careful comparison, to follow the instructions given.—Ed.]

An incident related at the late mass meeting in the interest of the Dental Protective Association is very appropriate here. An ardent practitioner, living in the distant part of New York State nine years ago, wrote to another in New York City inquiring what was thought of this new fad of crown- and bridge-work, which seemed to be exciting the ambitions of those who were determined to force themselves into the front rank on their merit. The answer came emphatically that no dentist of reputation was having anything to do with it. *Palma non sine pulvere.*

Evolution has played a successful part in this as in all things that have the germ of progress in them. I need not tell any intelligent, observing dentist that this branch of dental practice is now regarded as of immense importance. It has been of untold blessing to many, and will continue to be in the hands of competent

[1] The editor and publishers are not responsible for the views of authors of papers published in this department, nor for any claim to novelty, or otherwise, that may be made by them. No papers will be received for this department that have appeared in any other journal published in the country.

[2] Copyright, 1892, by Dr. C. M. Richmond.

practitioners. Many things have occurred on which to found a supposed basis of criticism, or more of fault-finding; and much wholesale condemnation has been indulged in, in which the principles upon which the work rests have been wholly disregarded. A vast amount of material has been given out that has been of an experimental nature, and, not infrequently, enthusiasm has gotten the better of good judgment, and hence many failures have marred the easy progress that would have occurred if a little slower pace had been made. Practical cases, worn for many months or years, carry a potent conviction to the minds of practical men, and commend the efficiency of such work. A detail of many such cases proves, by the clinical illustrations exhibited, that in each operation is indicated its own necessities and the mother of invention comes to the rescue, and each one results in a lasting benefit and success to the patient and a compliment to the operator. "Proving all things, and holding fast that which is good" is verified in this method of teaching, and must be so full of suggestion to one of limited experience as to study them with a diligence worthy of ultimate special ability in this branch of work. Only such as are eager to become able and have real mechanical talent can assure themselves that they have a mission in such a field of labor.

In this, my first article on crown- and bridge-work, I have taken an extreme case to show that portable bridge-work can be made a success if the work is well done. This case is an upper set of teeth held by two wisdom-teeth, and after seven years of continual use it shows no signs of failure.

The case was sent to me by Dr. LeRoy Satterlee, of the New York Dental College, and as he knew nothing of the conditions existing, had told the lady that she could have the case made without a plate. When the patient came to my chair she took from her mouth an upper set of teeth made on a rubber plate. I looked at her in surprise, and told her, as she had lost all of her upper teeth, nothing could be done for her. She looked up and said, "I have two new teeth," and with my glass I found the wisdom teeth erupting, and they had moved the plate until it had lost its suction. The patient, observing a doubt in my expression, looked up and said, "My heart is fixed on having a set of teeth put in without a plate, so don't say it can't be done."

An impression was taken and the patient sent home, and was told to come in at another time; this request being made to get time to think the case over.

After the patient had gone, a model was cast of fusible metal

Fig. 1.

Fig. 4.

Fig. 4.

Fig. 5.

Fig. 2.

Fig. 2.

Fig. 6.

Fig. 3.

Fig. 3.

Fig. 7.

for the case (shown in Fig. 1). Then the model was cut away with a burr to get more room to work at the gum line of the two teeth. I fitted two gold bands around them as best I could. When the patient came in again she was told of the chances of failure and of the poor chances for success; but she said, "Do the best you can." I then fitted the two bands to the teeth so closely that when the lower edges of the bands passed the largest place in the circumference of the teeth they fitted so tightly that they required the force of a pair of flat-nosed pliers to remove them. The bands were then cut the right length to open the mouth to a proper distance when they came in occlusion with the lower teeth. Swaged cusps were now soldered on to the bands. I had now two gold crowns which gave me my guide to work by, and afterwards I fastened them in place with cement.

The next step was to fit these two crowns with what I term telescopic fitting bands of clasp metal, No. 28 thick (United States standard). A plaster impression was then taken of the crowns and cast dies of fusible metal, as shown in Figs. 2 and 2, and there was now a fac-simile of each crown in a *hard* metal, which would endure all the hammering that might be required to perfectly fit the clasp bands to them, as shown in Figs. 3 and 3. In making these bands, I bent the clasp metal carefully to fit the dies, Figs. 2 and 2, as well as it could be done with clasp benders and pliers; then afterwards soldering them.

Now, with a copper hammer (which does not mar the surface of, the gold), the bands were driven on to the dies a little at a time, forcing the bands into place until they were perfectly fitted, as shown in Figs. 3 and 3.

An impression was now taken of the case in plaster, and I cast into it another fusible metal die so the two bands could be placed in position on the model, as shown in Fig. 4, which being done they were now ready for the "bite," which is taken in the ordinary way in wax.

The case was now ready for the teeth. After selecting a set which could be put into the case without the least grinding, I backed them up with pure gold and invested them, using half marble-dust and half plaster. Each tooth was then placed in its own investment, and they were put into the fire and heated up to the proper point for soldering. I then covered each one with solder, so that there was enough gold to make the bridge strong when the teeth were all put together in one piece. After the teeth came out of the fire they were ready to be waxed into place in the articulator, which now had the metal die with the bands in position, also the "bite" of the lower teeth. The teeth were then waxed in two pieces from the bands to "the centrals,"and invested again in two pieces, and when they had been cleaned free from the wax (which is done with boiling water) they were placed in the fire and soldered, each of the teeth together, and as they had been backed up before, it only required a small piece of solder to join each tooth. This was made in two pieces to avoid any shrinking of the parts after the *last* soldering, as it is only soldered in one place finally and the change is not perceptible, but if it is soldered all at once the case would not go on, as the contraction would bring the bands out of the circle towards the centre one-eighth of an inch or more. I was now ready to put the parts together, as in Fig. 5. As the centre of this bridge must be supported, I placed a small saddle, the size of half of a five-cent piece or the width of the centrals, on the gums in the centre of the case, as shown in Fig. 6. This is made by taking a piece of pure gold, thirty thick, of the right size and shape, and by burnishing it on to the centre of the model in the right position, a perfect-fitting saddle is secured. This was placed in the proper position on the cast, which was held in the articulator, and the two pieces, being soldered, were ready to slip on the die, Fig. 7, and the saddle was secured into position by wax, and the two halves were now held together by the same material, and the whole brought to form by wax, so it presents the same view as it does finished in Fig. 7. This was now strong enough to try in the mouth waxed together in the centre. If the case goes to its place, and the saddle is in its proper position, I take a plaster impression over the teeth to use as a trial matrix, as it is impossible to take the case off from the model, Fig. 5, or from the crowns in the mouth, without, in a slight degree, displacing the parts where it is waxed in the centre, and as the plaster impression is very perfect it can be used to try the case just before the last investment. Teeth were used in this case not gold tipped, as in *fixed* bridges, for the reason that if a tooth is broken, it can be at once repaired, and also because the artistic effect is perfect. Only one tooth has been broken in this case in the seven years it has been used.

I am in favor of *movable bridge*-work in *all* cases, as they can be easily and cheaply repaired. Drs. S. G. Perry, A. L. Northrop, and W. W. Walker have examined this case, and they can verify my assertion that it is as perfect as to mechanical and pathological conditions as if it were finished only yesterday.

The fusible metal used is as hard as zinc, and herewith I give the formula (metals to be melted together in the order named). This compound can be melted and poured into a plaster impression without generating steam, as it melts at 150° F.

| | |
|---|---|
| Tin | 20 parts by weight. |
| Lead | 19 " " " |
| Cadmium | 13 " " " |
| Bismuth | 48 " " " |

(To be continued.)

## Figures and Tables

**Fig. 1. Fig. 2. Fig. 3. Fig. 4. Fig. 5. Fig. 6. Fig. 7. f1:**